# *Ex vivo* tissue imaging for radiology–pathology correlation: a pilot study with a small bore 7-T MRI in a rare pigmented ganglioglioma exhibiting complex MR signal characteristics associated with melanin and hemosiderin

**DOI:** 10.1117/1.JMI.4.3.036001

**Published:** 2017-09-13

**Authors:** Kant M. Matsuda, Ana Lopes-Calcas, Michael L. Honke, Zoe O’Brien-Moran, Richard Buist, Michael West, Melanie Martin

**Affiliations:** a University of Manitoba, Max Rady College of Medicine, Department of Pathology, Rady Faculty of Health Sciences, Winnipeg, Manitoba, Canada; b Health Sciences Centre Winnipeg, Department of Pathology, Diagnostic Services of Manitoba, Winnipeg, Manitoba, Canada; c Memorial Sloan-Kettering Cancer Center, Department of Pathology, New York, New York, United States; d University of Winnipeg, Department of Physics, Winnipeg, Manitoba, Canada; e University of Manitoba, Max Rady College of Medicine, Department of Radiology, Rady Faculty of Health Sciences, Winnipeg, Manitoba, Canada; f University of Manitoba, Max Rady College of Medicine, Department of Neurosurgery, Rady Faculty of Health Sciences, Winnipeg, Manitoba, Canada

**Keywords:** ex vivo tissue imaging, radiology–pathology correlation, small bore 7-T magnetic resonance imaging, pigmented ganglioglioma, melanin-associated magnetic resonance imaging signal

## Abstract

To advance magnetic resonance imaging (MRI) technologies further for *in vivo* tissue characterization with histopathologic validation, we investigated the feasibility of *ex vivo* tissue imaging of a surgically removed human brain tumor as a comprehensive approach for radiology–pathology correlation in histoanatomically identical fashion in a rare case of pigmented ganglioglioma with complex paramagnetic properties. Pieces of surgically removed ganglioglioma, containing melanin and hemosiderin pigments, were imaged with a small bore 7-T MRI scanner to obtain T1-, T2-, and T2*-weighted image and diffusion tensor imaging (DTI). Corresponding histopathological slides were prepared for routine hematoxylin and eosin stain and special stains for melanin and iron/hemosiderin to correlate with MRI signal characteristics. Furthermore, mean diffusivity (MD) maps were generated from DTI data and correlated with cellularity using image analysis. While the presence of melanin was difficult to interpret in *in vivo* MRI with certainty due to concomitant hemosiderin pigments and calcium depositions, *ex vivo* tissue imaging clearly demonstrated pieces of tissue exhibiting the characteristic MR signal pattern for melanin with pathologic confirmation in a histoanatomically identical location. There was also concordant correlation between MD and cellularity. Although it is still in an initial phase of development, *ex vivo* tissue imaging is a promising approach, which offers radiology–pathology correlation in a straightforward and comprehensive manner.

## 1 Introduction

Due to its noninvasive nature, magnetic resonance imaging (MRI) has been playing a central role in medical management of central nervous system (CNS) disorders. Recent advances in multiparametric MRI expanded the potential for *in vivo* tissue characterization of pathologic changes in CNS diseases.^1^ As newer MRI methods emerge, it is crucial to verify that apparent abnormal features observed in imaging studies correlate with histopathology.

We began investigating *ex vivo* tissue imaging as a potential comprehensive approach for radiology–pathology correlation in order to bridge *in vivo* imaging findings and histopathology in a more straightforward fashion.^2^ A number of *ex vivo* imaging of brains from animal models have been conducted in the past.^3^^–^^11^ However, fewer studies involving human CNS disease have been reported, most of which employed autopsy brains.^12^^–^^14^

*Ex vivo* MRI of surgical specimens has been successfully performed on the human tissue from prostate cancer^15^ and breast cancer.^16^ A robust histopathology correlation was demonstrated by side-by-side comparison between *ex vivo* MRI and histopathology. In their studies, entire specimens from prostatectomy and breast mastectomy were imaged, which made subsequent correlation with *in vivo* MRI feasible when the specimens were properly oriented for both *ex vivo* imaging and histopathology preparation.

However, neurosurgical specimens of brain cancer pose a significant challenge in this regard. CNS tissue is very soft in consistency and easily fragmented during surgical resection such that identifying corresponding areas from *in vivo* MRI is extremely difficult. This is because CNS tissue consists of neurons, glial cells, and axons wrapped with myelin, without a solid stromal support. On the contrary, prostate and breast tissue consist of cohesive epithelial cells in the background of stroma with fibrous/fibroadipose tissue. Despite the disadvantages for correlation with *in vivo* imaging, MRI-histopathology correlation is a vital component in brain cancer management, because MRI-guided biopsy plays a central role in tissue diagnosis and molecular characterization for subsequent treatment planning.

In this study, *ex vivo* tissue imaging was incorporated to image fragments of surgically removed, human brain cancer tissue to demonstrate the feasibility of this approach.

We recently had a rare case of pigmented ganglioglioma, which exhibited unique paramagnetic properties associated with melanin and hemosiderin. Ganglioglioma (GG) is a low-grade glioneuronal neoplasm with incidence of 1.4% of all primary CNS neoplasms. Among them, pigmented GG is very rare, for which only five cases have been reported previously.^17^^–^^21^ Both pediatric and adult cases have been described. The former consists of desmoplastic variant with melanin pigments^17^^,^^18^ and the latter consists of those with melanin pigments^19^^,^^20^ and with hemosiderin pigments.^21^ In these case reports, the representative images from *in vivo* MRI were demonstrated to show overall appearances and the location of the lesions without detail MRI signal characteristics associated with melanin or hemosiderin.

Here, we demonstrated radiology–pathology correlation using *ex vivo* tissue imaging by a small animal 7-T MRI, in a case with rare pigmented GG containing both melanin and hemosiderin as a proof of concept in order to achieve point-by-point correlation at microscopic level in the neurosurgical specimens of brain cancer.

## 2 Materials and Methods

### 2.1 Case Information and In Vivo MRI

A 42-year-old previously healthy male presented with a first time seizure. CT scan demonstrated an intra-axial cortically based lesion with extensive calcification in the right parietal lobe. *In vivo* MR imaging with 1.5-T scanner (Avanto, Siemens) was performed with the following sequence parameters: T1-weighted image [Repetition Time (TR): 635 ms, Echo Time (TE): 17 ms, 512×448 matrix, slice thickness 5 mm], T2-weighted image (TR: 3200 ms, TE: 100 ms, 320×256 matrix, slice thickness 5 mm), susceptibility-weighted image (SWI) (TR 49 ms, TE 40 ms, 320×260 matrix, slice thickness 1.6 mm), and diffusion-weighted image (TR: 3012 ms, TE: 84 ms, 128×128 matrix, slice thickness 5 mm, b-value=0, 500, and 1000).

### 2.2 Histopathology Preparation for Diagnostic Pathology

After surgery, multiple fragmented pieces of tissue were received in formalin at the Department of Pathology, measuring ∼1.5×1.4×1.0  cm in aggregates. The majority of tissue was submitted for routine neuropathological examination for diagnostic purposes. Permanent paraffin-embedded tissue blocks were subsequently prepared for the diagnostic histopathologic examinations, hematoxylin and eosin (H&E) stain, Perl’s Prussian blue (PPB), Gomori Burtner silver impregnation method for melanin (MELN), von Kossa stain for calcium, and immunohistochemistry (IHC). The following panel of IHC was performed: glial fibrillary acidic protein (GFAP), synaptophysin, Neu-N, HMB45, MART-1, tyrosinase, P53 protein, and Ki-67 (Dako Canada, Mississauga, Ontario, Canada).

### 2.3 Specimen Preparation Prior to Ex Vivo Imaging

Pieces of tissue were collected from the surgical specimen, under the protocol approved by the institutional health research ethics board along with the consent obtained from the patient. The majority of tissue was submitted for routine neuropathological examination to render final diagnosis in a timely fashion within the clinically acceptable time frame in compliance with the approved protocol. Several small fragments were retained for research purpose and remained in formalin fixative until 7-T MRI was available for *ex vivo* tissue imaging. Among them, the two largest fragments were selected, which had been in formalin fixative for about 3 weeks. The remaining fragments were not used due to their small size (<1×1  mm). The first fragment was firm in consistency with a large heavily calcified nodule. The second one had a soft consistency similar to CNS tissue. The former was tentatively labeled as the tissue “A” and the latter as the tissue “B” (see Sec. 3 for details).

They were placed in distilled water overnight prior to imaging, in order to remove formalin as much as possible to minimize interference with MR signals on *ex vivo* imaging.^3^ Subsequently, they were placed in a small plastic container filled with 2% w/v (weight/volume) of agarose gel (Sigma-Aldrich, St. Louis, Missouri) in order to stabilize the tissue in the center position during imaging. Two markers were placed for specimen orientation using dried pasta, which was shown to be visualized with MRI without causing significant artifacts, and to be processed successfully with tissue for subsequent histopathologic tissue preparation. The first marker was long-cut pasta (Bucatini #6, DIVELLA, Rutigliano, Italy) prepared in 2.5 to 3 cm in length and placed in an upright position for *ex vivo* MRI. Imaging was conducted in a perpendicular direction to long axis of this marker. The second marker was short-cut pasta with the shape of an alphabet letter (ALPHABETS, ITALPASTA, Brampton, Ontario, Canada) in order to identify MR slices and corresponding histology sections with proper orientation. In this particular specimen, letter “G” was used to indicate “ganglio” or “glioma,” which was placed at the same level with the target tissue.

### 2.4 Ex Vivo Tissue Imaging with a Small Bore 7-T System

An *ex vivo* MRI scan was performed with a 21-cm bore 7-T MRI scanner (Bruker, Billerica, Massachusetts), with BG12 gradients, and with a maximum gradient strength of 448  mT/m using the following sequences: T1-weighted imaging: a RARE spin-echo sequence with TR 1200 ms, TE 11.6 ms, 8 echoes, 32 averages, 12 slices, imaging time 21 min 50 s, 300-μm slice thickness, and a 256×256 matrix, corresponding to 100  μm in plane resolution; T2-weighted imaging: a RARE spin-echo sequence with TR=6000  ms, TE=48  ms, 8 echoes, 12 averages, 12 slices, imaging time 25 min 36 s, 300-μm slice thickness, and a 256×256 matrix, corresponding to 100  μm in plane resolution; T2* (star) imaging: a FLASH gradient echo multiecho sequence with TR 775 ms, 12 echoes, TE 5 to 60 ms, 8 averages, 12 slices, imaging time 26 min 26 s, with the same geometry as T1 and T2; and diffusion tensor imaging (DTI): 7 diffusion-weighted images (DWIs), one without diffusion encoding gradients deemed to be the “b=0” image because the diffusion weighting b-factor is 0, and 6 with diffusion gradients in the standard tetrahedral gradient directions, each with TE=26  ms, TR=12,000  ms, 1 average, 12 slices, 300-μm slice thickness, 128×128 matrix corresponding to 200  μm in plane resolution for a total imaging time of 3 h and 25 min.

### 2.5 Histopathology Preparation for Postimaged Tissue

The postimaged tissue and the markers were processed together along with the surrounding agarose gel to maintain their position and spatial relation on the histology slides. Permanent paraffin-embedded tissue blocks and tissue sections with 6-μm thickness were prepared for the following stains: H&E stain, PPB, and MELN.

An initial histology section was stained with H&E method when the marker #2 was fully exposed on the glass slide to be recognized as an entire letter “G.” Due to difference in slice thicknesses between *ex vivo* MRI (300  μm) and histology sections (6  μm), 50 serial sections were subsequently obtained, several of which at different tissue levels (8 to 10 section intervals) were selected for H&E stain to assess morphology.

### 2.6 Image Analysis

After morphological assessment of histology sections, 12 slices from *ex vivo* MRI were evaluated to identify a representative one, which showed an overall morphology compatible with the majority of histology sections on visual inspection. We focused on a selected slice (slice #7) for subsequent image analysis and histopathologic correlation. Assessment for signal intensity for T1, T2, T2*, and mean diffusivity (MD) value on DTI was performed using a custom-written MATLAB^®^ (MathWorks, Natick, Massachusetts) script. For accurate construction of quantitative maps and ease of transferring regions of interest (ROIs) among images, the T1- and T2*-weighted images were registered in MATLAB^®^ to the T2-weighted image. The registration was performed using affine transformations as implemented by MATLAB’s^®^ registration function. ROIs were simultaneously drawn on all three images using a custom-built MATLAB^®^ script. The T1-weighted image was used as the initial reference. The ROIs were then checked for accuracy on the T2- and T2*-weighted images. The average and standard deviations of the intensities in all voxels in each ROI were calculated.

Each DWI was registered to the b=0 image using rigid transformations. The diffusion tensor, D, was estimated using a constrained weighted linear least squares fit. D was weighted to account for noise using Salvadaor weights [$w={\left(S/\sigma \right)}^{2}$].^22^^,^^23^ The Hermitian D tensor was reparameterized by a modified Cholesky factorization and thus constrained to be positive-definite.^24^ After diagonalization, the eigenvalues of D were used to calculate MD (mm2/s) for each voxel. MD scalar maps were created to visualize these data and perform ROI analysis with reference to histology.

Images of histology slides were obtained using an Olympus BX53 microscope with CellSens imaging software (Olympus Life Science, Tokyo, Japan), and digital slides were prepared using Aperio Scan Scope at 20× magnification (Leica Biosystems Inc., Buffalo, Grove, Illinois). Cell number was obtained by nuclear count on the digital slides using Aperio ImageScope software (Leica Biosystems Inc.). Nuclear count was used to define cellularity in a given ROI using the algorithm “Nuclear V9” from Image Scope. Detected nuclei on each ROI were carefully inspected with visual confirmation. The number of cells was manually corrected afterward.

Among 50 serial sections, histomorphology was assessed carefully on each selected tissue section in order to ensure that the overall tissue shape and size were consistent with the corresponding MR slice (slice #7). The histology sections from four different tissue levels (level 1, 11, 25, and 35) were selected for cellularity assessment. Four ROIs (500×500  μm) were randomly placed on the MD map. The corresponding ROIs were also placed on the digital histology slides in identical positions, and cellularity was counted. Subsequently, the average cellularity was multiplied by the number of interval sections (35 sections) in order to calculate an estimated number of the cells in a given ROI.

Next, the MD map was coregistered with the histology for correlation with MD value at a pixel level. A digital histology image with low-power magnification was prepared as a template, which included the tissue A and B, as well as the two markers in their entirety. A representative MD map (slice #7) was chosen to be aligned to this digital pathology image using a landmark-based registration and affine transformation. Landmarks were placed on both images using a custom-written MATLAB^®^ program.^25^ Four fiducial points were selected on the MD map, and the corresponding four fiducial points were then chosen on the digital pathology slide. The four points consisted of a single fiducial point, which was placed individually in each of the tissue A and B, as well as the marker #1 and #2. The program minimized the Euclidean distance between the landmarks of the two images and calculated the resulting two-dimensional affine transformation matrix. This transform was applied to the MD map, which was then cropped to the tissue of interest.

A total of 15 pixels with variable MD values were randomly selected. The corresponding ROIs (200×200  μm) were placed in a near-identical position on the digitized slides under the grid-guidance, and the number of cells was correlated with the MD value.

Subsequently, smaller ROIs with 100×100  μm were sequentially placed in order, which covered the entire tissue B, to demonstrate a cellularity map to qualitatively correlate with the MD maps. As described above, the average cellularity was obtained from the different tissue levels, and the estimated cell number was calculated in the same manner. The cell number in each ROI was entered in the Excel spreadsheet in a sequential order to generate the cellularity map. The approximate number of cells was further displayed in color-coded fashion to simulate the MD map. The color code consists of 11 different color gradients with the following order and corresponding cell number (see also Fig. 5 legend): white (0), light yellow (1 to 250), yellow (250 to 500), light brown (500 to 750), light orange (750 to 1000), orange (1000 to 1250), light red (1250 to 1500), red (1500 to 1750), dark red (1750 to 2000), brown (2000 to 2250), and dark brown (2250 to 2500).

## 3 Results

### 3.1 In Vivo MRI Findings

**Fig. 1:**
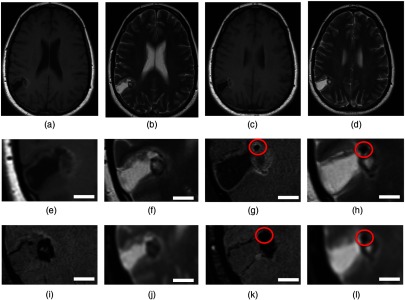
*In vivo* MRI findings. Representative images from two adjacent slices with the lesion on (a and c) T1-weighted image and (b and d) T2-weighted image in the upper panels. The magnified views of the lesion with each MRI sequence are shown in the lower panels: (e and g) T1-weighted image without contrast enhancement, (f and h) T2-weighted image, (i and k) SWI, and (j and l) ADC. The scale bar indicates 10 mm in actual length. (g, h, k, and l) A nodule with MRI signals characteristic for heavily calcification is indicated in red circles.

The MR imaging with 1.5 T demonstrated a solitary, primarily cystic, cortically based lesion in the right parietal lobe (Fig. 1). The cystic component of the lesion followed the cerebrospinal fluid signal on all sequences. The solid soft tissue nodule appeared heterogeneous in signal and demonstrates intrinsic T1-hyperintensity [Figs. 1(a), 1(c), 1(e), and 1(g)], with some areas of T2 hypointensity [Figs. 1(b), 1(d), 1(f), and 1(h)] and blooming on the susceptibility-weighted imaging sequence [Figs. 1(i) and 1(k)]. Some of these heterogeneous areas might correspond to the multifocal small calcifications. However, some of these intrinsic foci of T1-hyperintensity could also represent hemorrhage, or, to lesser probability, other paramagnetic properties such as melanin pigments. This nodule demonstrated minimal and faint enhancement in the postgadolinium sequences (data not shown). The apparent diffusion coefficient (ADC) map demonstrated heterogeneous values within the lesion [Figs. 1(j) and 1(l)]. In addition, there was a small nodule with an approximate size of 5×4  mm in diameter, which was devoid of signals on all sequences. It is reasonable to speculate that this nodule was likely to be heavily calcified without an intervening cellular component [circled in Figs. 1(g), 1(h), 1(k), and 1(l)].

### 3.2 Histopathologic Findings for Diagnostic Confirmation

**Fig. 2:**
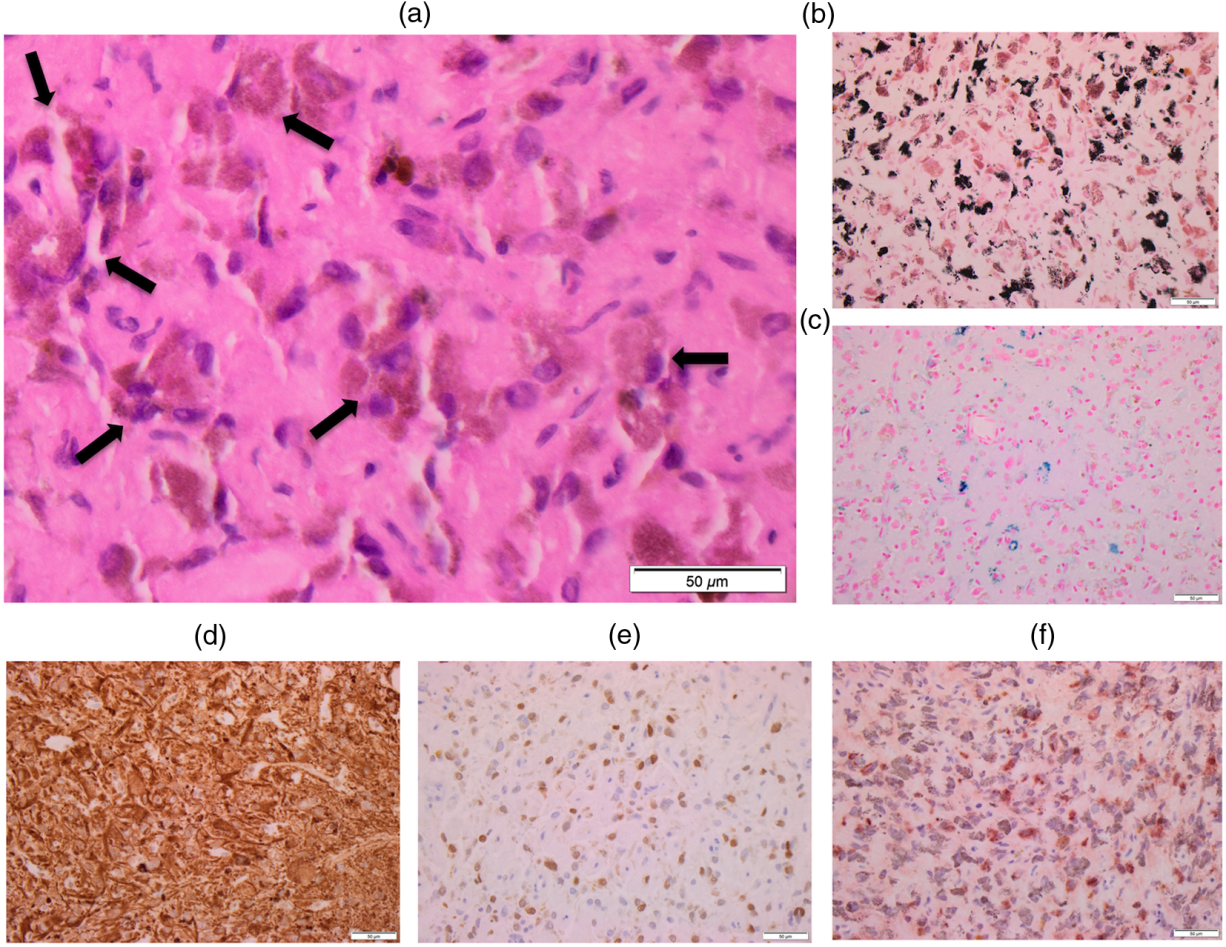
Histopathologic findings. (a) H&E stain with an enlarged image. Note many neoplastic cells with abundant brown pigments (allows). Special stains highlight melanin pigments in black (b) on MELN, and iron/hemosiderin pigments in blue (c) on PPB. IHC for (d) GFAP, (e) Neu-N, and (f) tyrosinase. At 400× magnification, the scale bar indicates 50  μm in actual length.

Because pigmented GG is very rare, we demonstrated histopathologic findings to confirm diagnosis. Histopathology from paraffin-embedded material showed fragments of tissue involved by the neoplastic cells composed of glial and neuronal components [Fig. 2(a)]. There were many neoplastic cells containing cytoplasmic brown pigments with variable sizes [Fig. 2(a), arrows]. No anaplastic features were noted, which include mitosis or necrosis. Histochemical stains revealed the pigments described above to be predominantly comprised by melanin (pigments stained in black) on the MELN [Fig. 2(b)], along with a minor component of iron/hemosiderin (pigments stained in blue), demonstrated on the PPB [Fig. 2(c)]. IHC revealed that the neoplastic cells were positive for glial marker [GFAP, Fig. 2(d)] and neuronal markers [Neu-N; Fig. 2(e), and synaptophysin; not shown]. Although melanocytic markers, including HMB-45 and MART-1, were negative (data not shown), many neoplastic cells showed strong immunoreactivity for tyrosinase [Fig. 2(f)], which is an enzyme that converts tyrosine to melanin, further supporting the evidence for melanin production.

Melanin has been shown to exhibit a unique signal pattern: hyperintense on T1-weighted image and hypointense on T2-weighted images,^26^ which is a useful MRI feature diagnostic of melanotic melanoma metastatic to the brain.^27^^,^^28^ However, care must be taken in this interpretation when there is accompanying hemorrhage, because acute/subacute hemorrhage can present a similar signal pattern.^29^ In addition, chronic hemorrhage with iron/hemosiderin depositions can potentially lower intensities on both sequences.

Histopathological correlation could retrospectively suggest that the rim of the nodule exhibiting higher T1- and lower T2-intensities may correspond to the area involved by pigmented ganglioglioma with abundant melanin. Although to lesser extent, there were iron/hemosiderin pigments which could potentially affect MR signals. Furthermore, there are multifocal calcifications that could also influence MR signals because calcification usually is devoid of MR signals due to lack of water content.

Because of the fragmented nature of the surgical specimen, it was challenging to correlate MRI signals with histology for melanin, iron/hemosiderin, or calcification with *in vivo* MRI as described before. Therefore, we performed *ex vivo* tissue imaging to achieve a comprehensive correlation with corresponding histology sections in anatomically identical manner.

### 3.3 Ex Vivo Tissue Imaging with Histopathologic Correlation

**Fig. 3:**
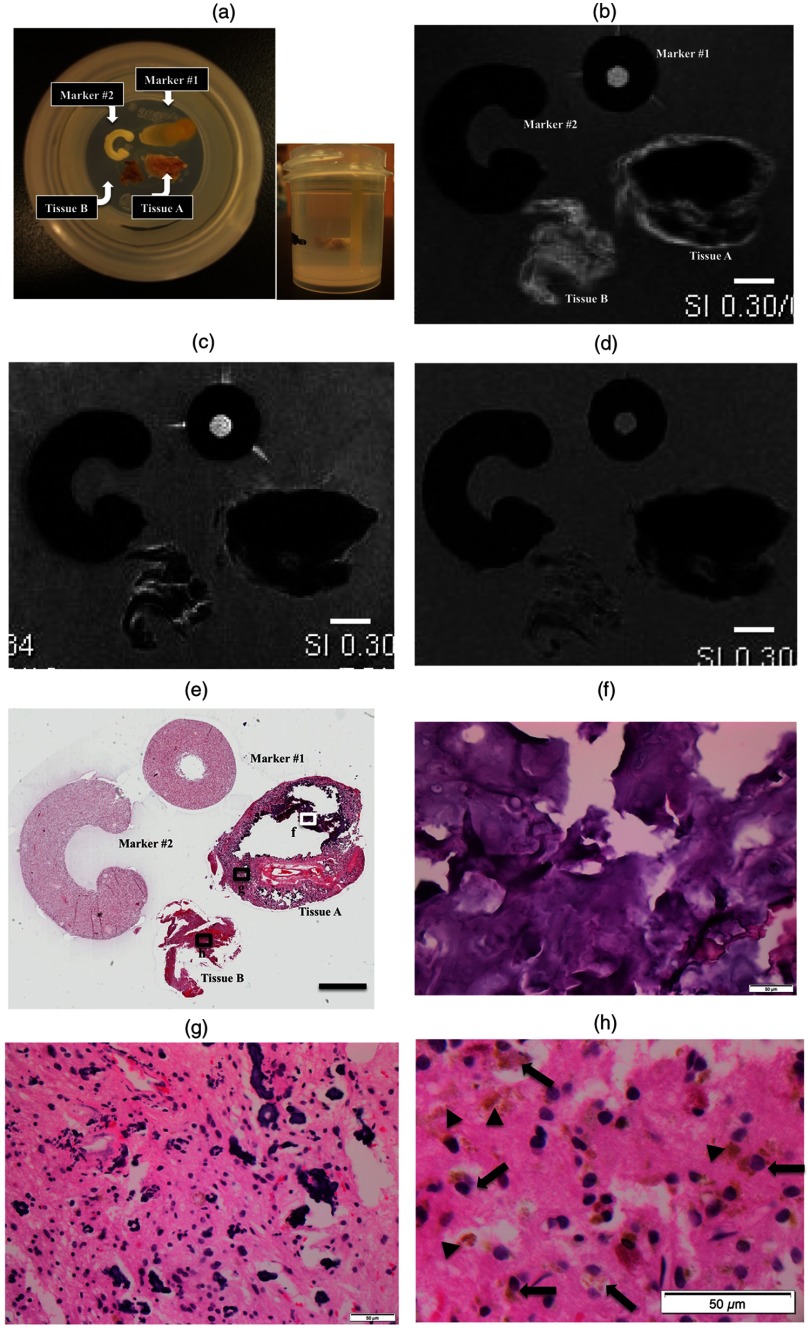
*Ex vivo* tissue imaging with histopathologic correlation. (a) Photographs of the specimen with markers in agarose gel. Representative slice (slice #7) from *ex vivo* tissue imaging with different MRI sequences: (b) T1-, (c) T2-, and (d) T2*-weighted images. The scale bar indicates 2 mm in actual length. (e) Corresponding histopathology: digital pathology with low-power view at 10× magnification. The areas from the tissue A (f) with dense calcification and (g) with admixed calcification and cellular component. (h) The tissue B with pigmented neoplastic cells without calcification with an enlarged image. At 400× magnification, the scale bar indicates 50  μm in actual length. The corresponding areas are highlighted in (e) with annotations: white rectangle with letter “f” for (f), black rectangles with “g” for (g) and “h” for (h). Note neoplastic cells with brown pigments (arrows) and pigmented depositions (arrowheads) in the tissue B (h).

Two fragments of tissue were collected for *ex vivo* tissue imaging. One fragment was firm in consistency, suggestive of containing a large calcified nodule, which was labeled as the tissue A [Fig. 3(a)]. The other was felt to be soft without grossly tactual calcification, which was labeled as the tissue B [Fig. 3(a)]. Based on similarity in size (∼5×4  mm in diameter), we selected the tissue A because this could correspond to the nodule with MRI characteristics consistent with heavy calcification, seen on *in vivo* MRI [Figs. 1(c), 1(d), 1(g), 1(h), 1(k), and 1(l)]. The tissue B was selected because it was unlikely to contain calcification, and thus ideal for histopathologic examination of the lesional tissue involved by pigmented GG. Calcification often compromises histopathologic examination for the following reason; calcified depositions are not able to be sectioned with the microtome and often create holes in the tissue sections. Histopathologic examination of such calcified tissue requires a prolonged fixation time with formalin and decalcification using a variety of mineral acids. Formic acid is used for decalcification in our department for routine clinical purpose. We did not decalcify the sample, because it often alters proteins and other molecules, which compromises subsequent IHC or histochemical stains.

A drawback for the tissue B could be that it was not possible to identify the corresponding area on *in vivo* MRI unlike the tissue A, because of the fragmented nature of neurosurgical specimens.

There were two markers placed in the agarose gel, which helped orienting the specimen for subsequent histopathological correlation [Fig. 3(a)]. We have been searching for appropriate markers in order to facilitate a quick and straightforward method for orientation of *ex vivo* imaging and histology sections.^2^ To achieve this goal, such markers should be able to be visualized with MRI without causing significant artifacts. They also have to be processed together with the tissue as permanent paraffin-embedded blocks. They have to be sectioned with a microtome, to remain on the glass sides and be stained to show their shape after various histochemical methods. We recently discovered that dried pasta is an ideal material, which meets our purpose. It absorbs water from the agarose gel and becomes soft to be readily sliced with a blade for sectioning. It remains intact after tissue processing and is able to be stained by the various histochemical staining methods on the glass slides. By placing the markers with different shapes in asymmetric positions next to the target tissue, it was easy to identify MR slices and corresponding histology sections with a proper orientation in top to bottom and right to left directions, respectively.

A representative image from *ex vivo* MRI was selected (slice #7), which showed a hyperintense rim on the T1-weighted image with homogenous hypointense center in the tissue A [Fig. 3(b)]. On the T2-weighted image, both areas showed hypointensity [Fig. 3(c)]. The susceptibility-weighted sequence, “T2*-weighted image,” further demonstrated a similar signal pattern [Fig. 3(d)]. These findings suggested that the outer rim contains melanin, and the center area was devoid of MR signals due to dense calcification without a cellular component. On visual inspection, corresponding histology confirmed a conglomerate of calcification in the center [Fig. 3(f)], and multifocal calcified clusters admixed with CNS tissue containing neoplastic cells in the rim [Fig. 3(g)]. The tissue B exhibited a pattern similar to the surrounding rim from the tissue A, which was compatible with MR signals associated with melanin. Many pigmented cells were recognized on H&E stain [Fig. 3(h)] in the corresponding area on visual inspection, which were histomorphologically identical to those seen in the diagnostic pathology slides [Fig. 2(a)].

### 3.4 T1/T2/T2* Signal Intensities and Correlation with Melanin and Iron

**Fig. 4:**
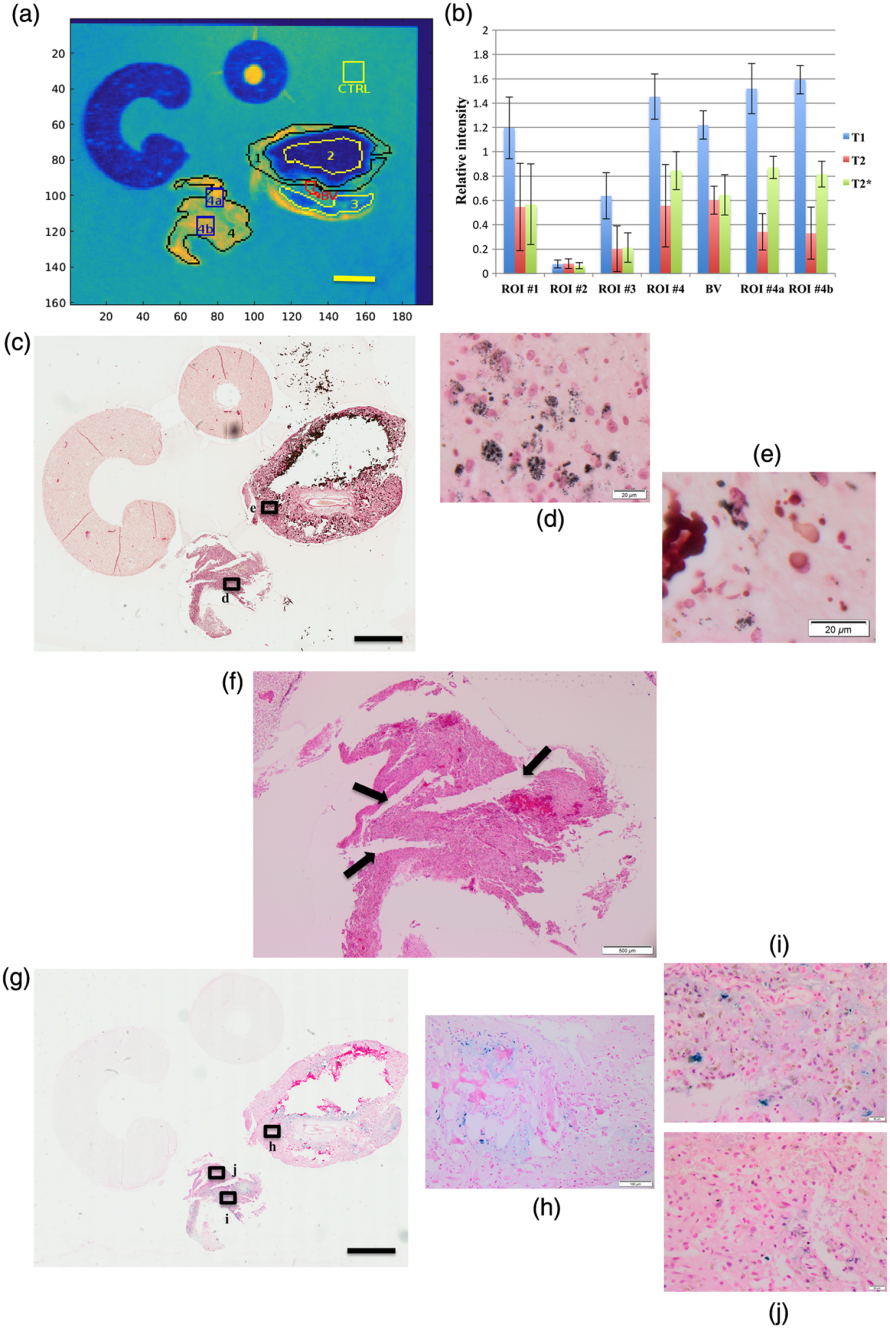
Image analysis for T1-/T2-/T2*-intensities with histopathologic correlation. (a) ROIs on T1-weighted image in pseudocolor map to highlight contrast. ROI #1 indicates hyperintense rim in the tissue A. ROIs #2 and #3 indicate hypointense areas within the tissue A. ROI#4 represents the entire tissue B in its entirely. ROIs#4a and #4b indicate sub-ROIs within the tissue B. The scale bar indicates 2 mm in actual length. (b) Relative signal intensity in each ROI with T1-/T2-/T2*-intensities. (c) T1-intensity in blue bar, T2-intensity in orange bar, and T2*-intensity in green bar. Melanin stain (MELN) with low-power view at 10× magnification. The scale bar indicates 2 mm in actual length. Magnified images from (d) the ROI #4 and (e) the ROI #1. The corresponding areas are highlighted in (c) with annotations: black rectangles with letter “d” for (d) and “e” for (e). At 600× magnification, the scale bar indicates 20  μm in actual length. (f) The cleft-like formations (arrows) within the tissue B. At 40× magnification, the scale bar indicates 500  μm in actual length. (g) Iron stain (PPB) with low-power view at 10× magnification. The scale bar indicates 2 mm in actual length. (h) The area with iron/hemosiderin depositions associated with dense calcification; at 200× magnification, the scale bar indicates 100  μm in actual length. The ROI (i) #4b and (j) #4a with different amount of iron depositions. At 600× magnification, the scale bar indicates 20  μm in actual length. The corresponding areas are highlighted in (g) with annotations: black rectangles with letter “h” for (h), “i” for (i), and “j” for (j).

Next, we investigated the effect on MR signals by different paramagnetic molecules, such as melanin and iron/hemosiderin through image analysis in a semiquantitative fashion. Multiple ROIs were placed based on the T1-weighted image, shown as a pseudocolor map to highlight the contrast [Fig. 4(a)]. The ROI #1 represented an area with hyperintense rim, and the ROIs #2 and #3 represented hypointense areas in the tissue A. The ROI #4 represented the tissue B, which contained no calcification. There was a single ROI with exception, which was based on a hyperintense ring-like area on T2-weighted image, labeled as “blood vessel (BV).” On visual inspection, the corresponding histology demonstrated fibroconnective tissue surrounding a medium-sized BV without pigmented neoplastic cells. The control ROI was placed within the agarose gel for reference, labeled “control (CTRL).” Average voxel intensity was normalized with that of the reference ROI (CTRL) on all three sequences, respectively [Fig. 4(b)]. The unpaired t-test was performed among the ROIs, and the results were summarized in Table 1.

**Table 1:** Statistical analysis with unpaired t-test.

	T1	T2	T2*
#1 versus #2	p<0.0001*	p<0.0001*	p<0.0001*
#1 versus #3	p<0.0001*	p<0.0001*	p<0.0001*
#1 versus #4	p<0.0001*	p=0.5067	p<0.0001*
#2 versus #3	p<0.0001	p<0.0001*	p<0.0001*
#2 versus #4	p<0.0001*	p<0.0001*	p<0.0001*
#3 versus #4	p<0.0001*	p<0.0001*	p<0.0001*
#1 versus BV	p=0.5116	p=0.2535	p=0.0846
#2 versus BV	p<0.0001*	p<0.0001*	p<0.0001*
#3 versus BV	p<0.0001*	p<0.0001*	p<0.0001*
#4 versus BV	p<0.0001*	p=0.3245	p<0.0001*
#4a versus #1	p<0.0001*	p<0.0001*	p<0.0001*
#4b versus #1	p<0.0001*	p<0.0001*	p<0.0001*
#4a versus BV	p<0.0001*	p<0.0001*	p<0.0001*
#4b versus BV	p<0.0001*	p<0.0001*	p<0.0001*
#4a versus #4b	P=0.0014*	p=0.6371	p<0.0001*

* Statistically significant.

The bold values represent statistically not significant.

Signal intensities were significantly lower in the ROI #2 on both T1- and T2-weighted images, compared with other ROIs (p<0.0001), which can be explained by dense calcification without any cellular component on H&E [Fig. 3(f)]. The ROI #4 demonstrated T1-hyperintense and T2-hypointense [Fig. 3(c)], the pattern of which was compatible with the MR signal associated with melanin. The melanin stain (MELN) confirmed the presence of abundant melanin pigments in the corresponding area on visual inspection [Fig. 4(d)]. The ROI #1 exhibited a similar pattern, which corresponded to the hyperintense rim from tissue A. However, image analysis revealed no statistically significant difference with BV on all three sequences: T1- (p=0.5116), T2- (p=0.2350), and T2*-intensities (p=0.0846). There were scattered black melanin pigments in the corresponding area on the MRLN stain with visual inspection [Fig. 4(e)]. This likely indicated that the amount of melanin was only minimal in the ROI #1, which was not sufficient to alter signal intensity.

In the ROI #3, intensities on all three sequences were intermediate between the ROI #2 and the ROI #1, the differences of which were statistically significant. This may reflect relative proportion of calcification to the cellular component [Fig. 3(g)].

In tissue B, significant differences were observed on T1-intensity between the ROI #4 and all ROIs from tissue A, which supported the paramagnetic property of melanin. On T2-signal, however, T2-intensity was statistically different with the ROIs #2 and with #3 (both p<0.0001) but not with the ROI #1 (p=0.5067) or with BV (p=0.3425). On the T2-weighted image, there were hyperintense narrow bands running through the tissue B, which could be a likely explanation. In histology, the corresponding areas did not demonstrate overt morphological changes or tissue with different composition, such as fibroconnective tissue. However, careful observation on histology suggested that there were cleft-like formations in the corresponding areas [Fig. 4(f)]. As such, it is reasonable to consider that these T2-hyperintense bands may reflect penetration of agarose gel, which focally involved the tissue.

Therefore, we investigated further using additional smaller ROIs, labeled “#4a,” and “#4b,” within the tissue B [Fig. 4(a)]. They were rectangular and placed within the areas containing minimal T2-hyperintense bands. They demonstrated lower T2-intensity [Fig. 4(b)] and were statistically significant compared to both with BV and the ROI #1.

Regarding the T2*-weighted image, the PPB stain for iron demonstrated a heterogeneous distribution of iron/hemosiderin pigments within both tissue A and B [Fig. 4(g)]. In the tissue A, iron/hemosiderin pigments appeared to be closely associated with the calcification [Fig. 4(h)], which could alone significantly lower T2*-intensity. In contrast, robust pigments were seen in the tissue B, which contained no calcification. By visual evaluation, many iron/hemosiderin pigments were seen in the ROI #4b [Fig. 4(i)], while they were minimal in the ROI #4a [Fig. 4(j)]. Image analysis demonstrated significantly lower T2*-intensity in the ROI #4b, compared with the ROI #4a (p<0.0001), which was concordant with visual impression. Interestingly, T1-intensity was significantly lower in the ROI #4a (p=0.0014) despite no difference on T2-intensity. This may raise the question of whether iron can affect the paramagnetic property of melanin on T1-signal. However, it is premature to conclude without quantifying melanin, which is challenging to perform in a reliable manner on formalin fixed paraffin-embedded tissue.

### 3.5 Mean Diffusivity for Correlation with Cellularity

**Fig. 5:**
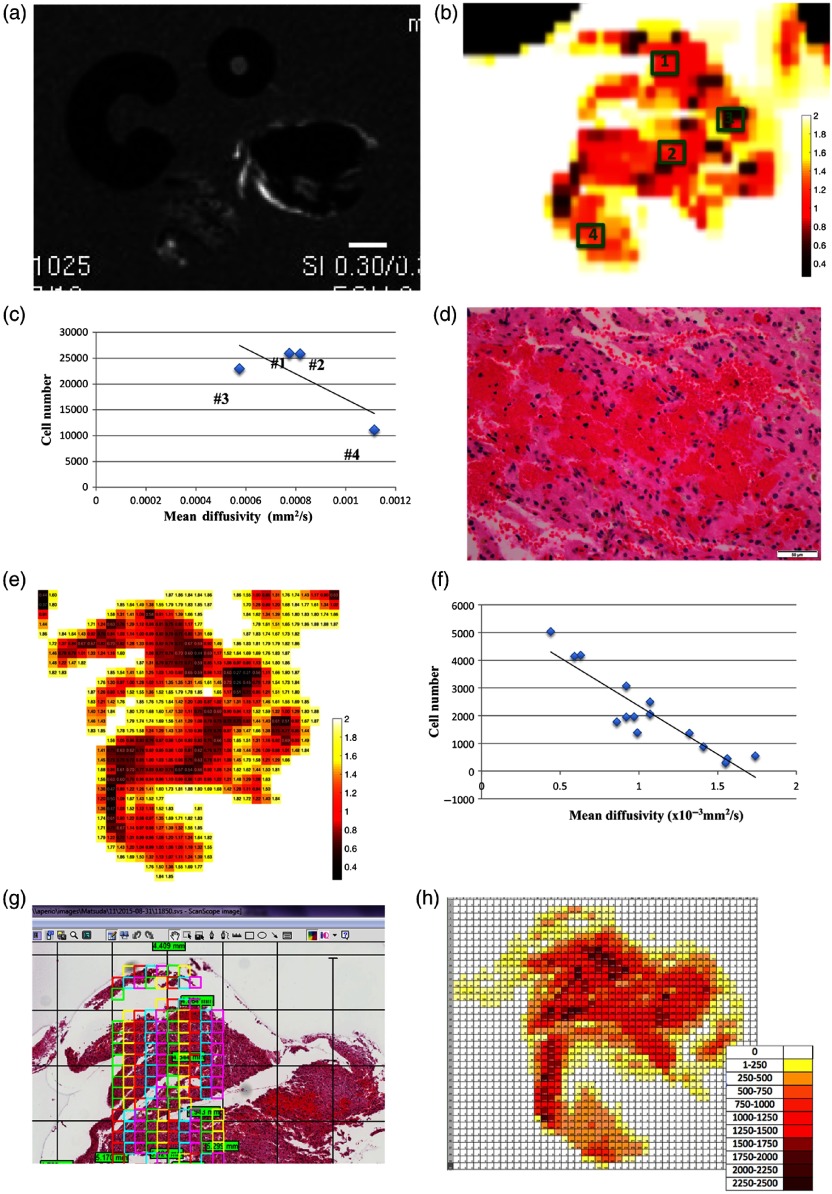
(a) MD map for correlation with cellularity. DWI in grayscale. (b) Pseudocolor-coded MD map from tissue B. The scale bar indicates 2 mm in actual length. Four ROIs with 500×500  μm are highlighted in green. (c) Correlation between cellularity and MD value in the ROIs #1 to 4. The estimated number of cells was calculated by the average from four different histology sections with different tissue levels, multiplied by the number of interval sections (35 sections). (d) Histopathology corresponding to the ROI #3 showed tight and dense clusters of RBCs. At 400× magnification, the scale bar indicates 50  μm in actual length. (e) The pseudocolor-coded MD map of tissue B after coregistration with histology. The MD values (${\mathrm{mm}}^{2}/s$) were multiplied by 1000 times for demonstration purpose. (f) Correlation between the MD values from 15 selected pixels and cellularity from the corresponding ROIs with 200×200  μm. (g) Sequentially placed ROIs with 100×100  μm on the digital pathology slide. (h) Qualitative correlation between the MD map and the cellularity map generated from the smaller ROIs with 100×100  μm. The estimated number of cells was calculated as described previously.

We further explored a correlation with DTI. Specific features characteristic for GG in DTI have not yet been established. ADC maps often show slightly increased or decreased values in GG,^30^ which have been speculated to reflect cellularity. Our case also demonstrated a heterogeneous pattern with variable ADC values [Figs. 1(j) and 1(l)]. The MD map was generated from DTI data, which corresponds to the ADC map in *in vivo* MRI. In grayscale image, the tissue B exhibited a heterogeneous pattern [Fig. 5(a)]. The MD map of the tissue B was further displayed in pseudocolor [Fig. 5(b)]. ADC value has been reported to correlate inversely with cellularity.^31^ To investigate if the MD value reflects cellularity, the cell number was counted by image analysis on the digitized slides. Nuclear count was used to define cellularity in a given ROI using the computer software, “Image Scope.” Previous studies indicated that paraffin processing usually causes shrinkage of tissue up 20%^32^^,^^33^ due to complete dehydration from tissue. As such, multiple sections were selected from four different tissue levels (levels 1, 11, 25, and 35) among 50 serial sections, morphology of which was in keeping with the corresponding selected MRI slice (slice #7).

Four ROIs with the size of 500×500  μm were randomly placed [Fig. 5(b)], which demonstrated a negative correlation [Fig. 5(c)]. Among four ROIs, however, MD value from the ROI #3 was somewhat disproportionally low, compared with cellularity. In the histology image [Fig. 5(d)], there was a hemorrhagic focus with tight and dense clusters of red blood cells (RBCs) in the corresponding area. Image analysis did not recognize an RBC as a single cell, because it lacks a nucleus. However, diffusion could be restricted within the clusters of RBCs in a similar manner as high cellularity areas.

Next, we further correlated cellularity with MD value at a single-pixel level. To perform such correlation, the MD map was coregistered with the histology [Fig. 5(e)]. Subsequently, we selected 15 pixels with variable MD values and placed the corresponding ROI with the size of 200×200  μm in a near-identical location on the digital histology slide. The estimated number of cells was calculated from the images with different tissue level as described above. There was a negative correlation with a coefficient value of 0.9146 [Fig. 5(f)].

Finally, we attempted to demonstrate a correlation in a qualitative manner, using smaller multiple ROIs with the size of 100×100  μm [Fig. 5(g)]. The approximate number of cells was further displayed in color-coded fashion to simulate the MD maps [Fig. 5(h)]. Initially, we performed the cell counts using ROIs, which had the same size (200×200  μm) as the pixels in the DTI. However, we found the subset of ROIs, especially those at the edge of tissue, included an area with significant empty space, which produced a seemingly unclear edge. In contrast, smaller ROIs, which we used for our analysis, were able to provide a better image with less interference by such empty spaces [Fig. 5(g)]. Although not a perfect match, overall, the cellularity map demonstrated a similar pattern, which was concordant with the MD maps [Figs. 5(e) and 5(h)].

### 3.6 Discussion

We demonstrated the feasibility of our approach as a pilot study for radiology–pathology correlation using *ex vivo* tissue imaging in a rare pigmented GG, which contained a unique combination of different types of pigments.

This was the sixth case of pigmented GG.^17^^–^^21^ In the previous three adult cases,^19^^–^^21^ two had pigments only of melanin^19^^,^^20^ and the remaining one had that of iron/hemosiderin.^21^ Because they were case reports focusing on the histopathologic findings of this rare subtype of GG,^17^^–^^21^ MR signal characteristics associated with melanin or hemosiderin were not specifically described, and radiology-pathology correlation was not discussed.

In contrast, both melanin and iron/hemosiderin were present in our case. The former was a predominant component over the latter. Additionally, tyrosinase expression further supported melanin production in the neoplastic cells. Clinical *in vivo* MRI exhibited a complex signal pattern, making it difficult to suggest the presence of melanin on imaging grounds alone. This was particularly challenging due to concomitant calcification that was devoid of MR signals. Because the pigmented subtype is unlikely to influence the WHO grading for prognosis, melanin-associated MR features may have little impact on clinical management. However, it is of great interest from MR physics perspective to study the difference in MR signals via radiology–pathology correlation, in such a rare case exhibiting multiple molecules with different paramagnetic properties.

As described previously, GG is not common with overall incidence of <2% of all intracranial primary CNS neoplasm. Pigmented GG is even far less, with only five cases reported previously. Therefore, only a single case was investigated as a pilot study for *ex vivo* tissue imaging.

Due to their fragmented nature of the neurosurgical specimens, it was difficult to identify corresponding regions in *in vivo* MRI. In addition, correlation was even more challenging because of the heterogeneous and intermixed distribution of different paramagnetic molecules in this case. This prompted us to explore a straightforward approach, which can offer point-by-point correlation in anatomically identical locations for histopathological validation of MR signal characteristics. To overcome this problem, we incorporated *ex vivo* tissue imaging with subsequent image analysis for both MRI and histopathology. The areas with abundant melanin exhibited a characteristic melanin-associated signal pattern. Signal intensities were sharply decreased in the areas with melanin and concomitant calcification, which demonstrated a similar MR signal profile in the areas with dense conglomerate calcification alone. Even a small amount of iron from hemosiderin lowered the intensity on SWI, while T1- and T2-intensities associated with melanin appeared not significantly altered. By side-by-side correlation between *ex vivo* MRI and corresponding histology, a variety of different paramagnetic molecules were successfully demonstrated in histoanatomically identical locations that correspond to areas with specific MR signal profile. In addition, MD value from DTI was shown to closely reflect cellularity between *ex vivo* imaging and histopathology.

**Fig. 6:**
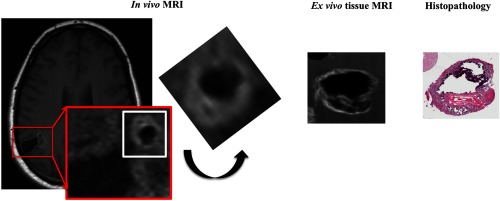
Correlation among *in vivo* MRI-*ex vivo* MRI-histopathologic correlation targeting on the tissue A with heavy calcified nodular formation (rectangle in white). In the magnified view of the area with red rectangle, a nodule with MR signal characteristics for heavy calcification is demonstrated, which is most compatible with the tissue A in *ex vivo* MRI and histopathology. On *in vivo* MRI, the area highlighted in a white square is cropped and rotated in a position that provides a similar appearance with *ex vivo* MRI and histopathology. Correlation for the tissue B is difficult due to fragmented nature of the neurosurgical specimen, respectively.

As shown as a schematic presentation in Fig. 6, we made our best effort to attempt correlation between *in vivo* MRI and *ex vivo* MRI and histopathology focusing on the tissue A with a heavily calcified nodule. Based on the size, as well as MRI signal characteristics, this nodule seen in *in vivo* MRI is most consistent with the tissue A on *ex vivo* MRI and histopathology. On the contrary, the tissue B could be any portion of the soft tissue tumor nodule on *in vivo* MRI, and precise correlation is limited due to the fragmented nature of the neurosurgical specimens. However, this approach appears to have the potential to translate *in vivo* MRI findings to the underlying histopathological findings, especially in neurosurgical specimens where tissue fragmentation is difficult to avoid.

Further challenges remain for future applications. One of them is the development of an integrated image processing platform with advanced coregistration, where ROIs can be placed simultaneously on the images from both *ex vivo* tissue imaging and digital pathology in a MATLAB^®^-based image analysis tool. This is a critical step to conduct precise point-by-point correlation. Second is how to translate MD values from *ex vivo* data into ADC values from *in vivo* DTI. Because different resolutions, temperature, and potentially different pulse sequence parameters are used, it may not be feasible to directly compare value between the two. Alternatively, it could be reasonable to compare the standardized value, which is, for example, normalized to uninvolved CNS tissue. In this particular case, the specimen did not contain such normal CNS tissue. For this purpose, *ex vivo* imaging of the interface area containing normal CNS tissue may be a potential option. Such an interface area can be sampled from a relatively large resection or an autopsy specimen with brain cancer. Third is in regard to correlation between DTI and cellularity, where another potential challenge arises when this application is further expanded. It has been reported that the cellularity is not the only factor that influences ADC value. For example, ADC has been speculated to also reflect cellular death, such as necrosis or apoptosis,^34^ because DTI measures the diffusivity of water molecules that interact with the different cellular constitutes and reflect disintegration of cellular structures caused by cellular death. In this particular situation, our case was a low-grade neoplasm, which usually does not harbor tissue necrosis or apoptosis spontaneously. In keeping with this feature, no necrosis or apoptosis was recognized in our case such that cellularity was likely a major factor contributing to the ADC value. However, cellular necrosis and apoptosis are frequently seen in more aggressive neoplasms, such as glioblastoma for which, pseudopalisading necrosis is a hallmark of diagnostic criteria. In histopathology, this often exhibits multifocal microscopic foci admixed with viable neoplastic cells. In this setting, *ex vivo* tissue imaging using high-field MRI can offer a potential approach,^35^ because it can provide higher resolution at a microscopic level, as also demonstrated in this study.

Regarding correlation for MR signals associated with concomitant melanin and hemosiderin, quantification of these molecules is of interest for our future investigation. Potential methods may include spectrophotometry, chemical degradation, or high performance liquid chromatography.^36^ There is also a study using florescent spectroscopy in a recent publication.^37^ However, these technologies require homogenization of tissue samples such that it may not be the best approach to achieve histopathology correlation in anatomically identical locations as shown in our study. Imaging mass spectrometry is certainly a potential alternative method.^38^ But this technology requires frozen tissue. Since pigmented GG is very rare, it is reasonable to target more common brain cancers with relatively abundant tissue available for both clinical and research purposes. Possibly, any cases with metastatic melanoma to the brain could be good candidates for this purpose. We had several cases in these recent years. Unfortunately, they were all amelanotic melanoma lacking melanin pigments. But this will be certainly reserved as a potential target for future investigation.

The scope of current biomarkers has been expanded to include medical imaging. When the imaging features are demonstrated to reflect pathology at the cellular and molecular level, imaging biomarkers will become a powerful and reliable biomarker in clinical management.^39^ In conclusion, our approach underscores the utility of *ex vivo* tissue imaging for the establishment of radiology–pathology correlation, which has a potential to deliver a unique platform for the interface between radiology and pathology. It will enhance imaging biomarker development, which will expand the diagnostic applications of MRI and medical imaging research.
